# Intramedullary non-specific inflammatory lesion of thoracic spine: A case report

**DOI:** 10.1186/1477-7819-8-3

**Published:** 2010-01-15

**Authors:** Alessandro Landi, Valerio Di Norcia, Demo Eugenio Dugoni, Roberto Tarantino, Martina Cappelletti, Manila Antonelli, Antonio Santoro, Roberto Delfini

**Affiliations:** 1 Department of Neurosurgery, University of Rome Sapienza, Rome, Italy; 2 Department of Pathological Anatomy, University of Rome Sapienza, Rome, Italy

## Abstract

**Background:**

There are several non-neoplastic lesions which mimick intramedullary spinal cord neoplasm in their radiographic and clinical presentation. These can be classified as either infectious (TB, fungal, bacterial, parasytic, syphilis, CMV, HSV) and non-infectious (sarcoid, MS, myelitis, ADEM, SLE) inflammatory lesions, idiopathic necrotizing myelopathy, unusual vascular lesions and radiation myelopathy. Although biopsy may be indicated in many cases, an erroneous diagnosis of intramedullary neoplasm can often be eliminated pre-operatively.

**Case description:**

the authors report a very rare case of intramedullary non-specific inflammatory lesion of unknown origin, without signs of infection or demyelinization, in a woman who showed no other evidence of systemic disease.

**Conclusions:**

Intramedullary lesions that mimick a tumor can be various and difficult to interpret. Preoperative MRI does not allow a certain diagnosis because these lesions have a very similar signal intensity pattern. Specific tests for infective pathologies are useful for diagnosis, but histological examination is essential for establishing a certain diagnosis. In our case the final histological examination and the specific tests that we performed have not cleared our doubts regarding the nature of the lesion that remains controversial.

## Background

There are several non-neoplastic lesions which mimick intramedullary spinal cord neoplasm. These can be classified as either infectious (TB, fungal, bacterial, parasytic, syphilis, CMV, HSV) and non-infectious (sarcoid, MS, myelitis, ADEM, SLE) inflammatory lesions, idiopathic necrotizing myelopathy, unusual vascular lesions and radiation myelopathy. Although biopsy may be indicated in many cases, an erroneous diagnosis of intramedullary neoplasm can often be eliminated pre-operatively.

## Case report

**Figure 1:**
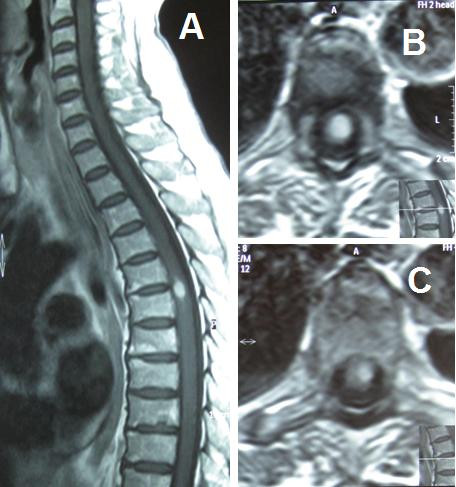
**Preoperative MRI: sagittal (a) and axial (b/c) T1 weighted image with contrast showing the intramedullary gadolinium-enhancing lesion at level T5-T6**.

A 71-year-old Italian woman presented a 2-month history of numbness and pain involving the left leg. She underwent orthopedic evaluation and articular ankle echography for the diagnostic suspicion of Baker cyst, that were negative. She also underwent lumbosacral MRI that did not show any signs of degenerative or traumatic injuries. One month later she developed radicular pain in both legs with hypoesthesia. She underwent cervico-dorsal MRI with contrast that showed a gadolinium-enhancing lesion within the spinal cord at T5-6 with maximum diameter of 11 mm. In the axial sequences the lesion seemed to be completely intramedullary without any signs of bulging. The neuroradiological aspects of the lesion were interpreted as an intramedullary astrocytoma or ependymoma. Neurologically, she had symmetric tendon reflexes, exaggerated in the legs, Babinsky sign on both legs, moderate paraparesis, hypoesthesia and dysesthesia of the entire left leg and left thorax below the T5 metamer. One month after the first MRI, during recovery, she underwent another dorsal MRI with contrast, which confirmed the presence of this intramedullary gadolinium-enhancing lesion at level T5-T6: this was interpreted as hemangioblastoma or ependymoma but, according to the neuroradiologist, it was impossibile to exclude other diagnostic hypothese (fig.1). As a matter of fact, the worsening of neurological symptomatology, expecially the progression of paraparesis, persuaded us to adopt a decompressive surgical strategy and so the patient underwent surgical treatment. A T5-T6-T7 laminectomy was performed, and the dura was opened. There was no evidence of extramedullary abnormality. Posterior longitudinal myelotomy was performed and a well-circumscribed grayish-red lesion was exposed. A histological sample for the extemporary and definitive examination was taken, which showed a histological pattern of small-cell tumoral lesion. For this reason a complete removal with CUSA was performed.

**Figure 2:**
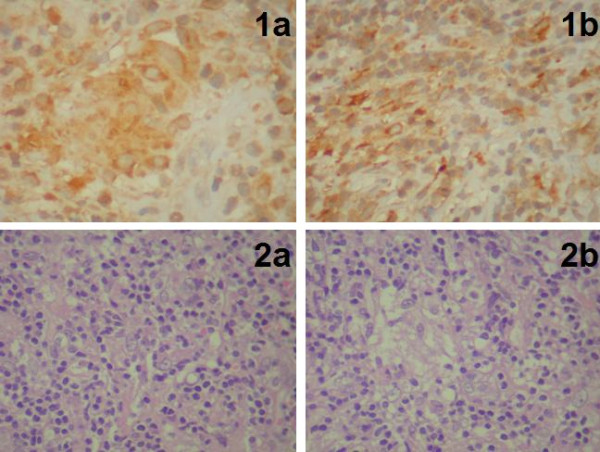
**1a/b Cytoplasmic immunoreactivity for CD68 is evident**. **2a/b **-The lesion is composed of a mixture of lymphocytes with plasmacells and macrophages. In figure b a granulomatous reaction is evident.

**Figure 3:**
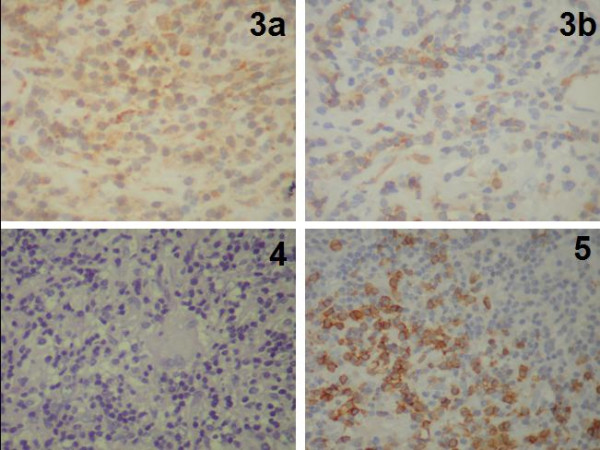
**A CD 68 reactivity - 3b Surface immunoreactivity for CD3**. 4/5 ziehl- Neelsen reactivity and Surface immunoreactivity for CD20

**Figure 4:**
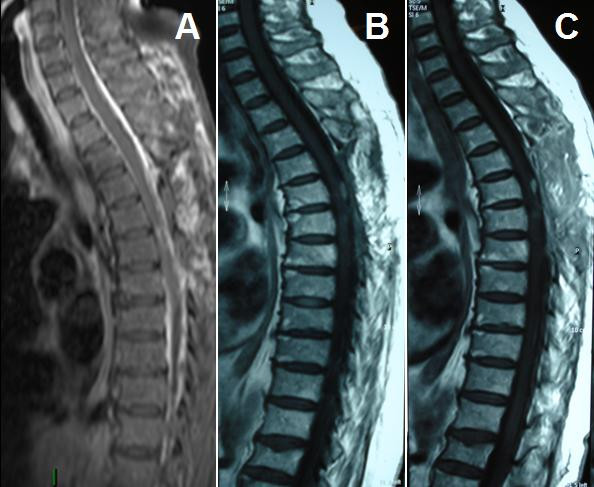
**Postoperative MRI: Sagittal T1 weighted image with gadolinium 10 days (a), 3 months (b), and 10 months (c) after surgery**.

The definitive histological examination of the specimen revealed an inflammatory lesion which was composed of a mixed infiltration of mature B and T lymphocytes, with plasma cells and macrophages. Abundant vascular channels, often with hyperplastic endothelium, and focal fibroblastic reaction were observed. The macrophages were occasionally organized to form granulomas. This mass of non-neoplastic inflammatory cells of unknown origin was also studied using histochemical technique (PAS and Ziehl Neelsen), but no fungal or bacteria were found. Also the non-neoplastic nature of the lesion was demonstrated by immunohistochemical studies, which confirmed the mixed nature of lymphocytes, and by the polyclonality of plasma cells with positivity for kappa and lambda light chains of immunoglobulins (fig 2, 3). Postoperatively, there was a complete regression of radicular pain and paresthesia in the left thorax and leg. Postoperative dorsal MRI with contrast was performed 10 days after surgery and confirmed complete removal of the lesion without any signs of residual disease (fig.4a). Postoperative laboratory and radiological exams was performed, such as Toxotest, BK test and Chest RX for the diagnostic hypothesis of toxoplasmosis, TBC, histiocitosys X, or sarcoidosis, that were all negative. We also performed the liquor level of cerebrospinal fluid angiotensinconverting enzyme (ACE) in suspected neurosarcoidosi, which showed a value of 3.2 nmol/mL/min, non discriminatory. The patient was discharged on the seventh postoperative day. At follow up thoracic MRI with contrast was performed 3 (fig.4b) and 10 months (fig.4c) after surgery, and did not show any signs of disease.

## Discussion

We will discuss in detail the differential diagnosis we considered:

### Neoplastic lesions

Intramedullary tumours of the spinal cord are rare. The most common are astrocytomas and ependymomas which together account for 90%. These lesions can cause significant difficulties in the differential diagnosis between inflammatory diseases such as multiple sclerosis (MS) and acute disseminated encephalomyelitis (ADEM), and vascular abnormalities and neoplasms. Because the clinical characteristics of neoplastic and non-neoplastic spinal cord lesions may be very similar, we rely on MRI for making a correct diagnosis. The MRI makes it possible to locate tumours in the extradural, intradural or extramedullary spaces, or within the cord itself; the tumour's location and its MRI characteristics may actually identify its specific type. In some instances, however, it is quite difficult to identify the exact nature of the pathological changes without a complete and detailed history and clinical examination [1]. In addition, due to the extreme heterogenity of the symptoms and radiological aspects of these lesions, which causes many difficulties in differential diagnosis, it's very important to perform a histological examination, and an extemporary histological finding during surgery. In our case the extemporary histological finding oriented us towards a small cell tumoral lesion, guiding our surgical strategy towards a total removal instead of a biopsy. The most common intramedullary tumors are astrocytomas and ependymomas. Cytological analysis of our lesion did not show the presence of glial-type tumoral cells. In addition, the non-neoplastic nature of the lesion was confirmed by isolation, using immunohistochemical techniques, of T and B lynphoid cells, with the individuation of a polyclonality of plasma cells and with the evidence of slight kappa and lambda chains of immunoglobulins. This histological pattern indicated the possibility of a granulomatous inflammatory intramedullary lesion.

### Granulomatous inflammatory and infectious lesions

Granulomatous lesions affecting the spinal cord are principally tubercolosis, sarcoidosis, brucellosis and histocytosis X [2]. In our case the postoperative performance of a BK test and toxotest excluded the possibility of TBC [3] and toxoplasmosis [4]. Postoperative radiological investigations like chest X-ray, excluded presence of extramedullary localizations of histiocytosis X [5] and sarcoidosis [6]. As a matter of fact, our first diagnostic hypothesis, was sarcoidosis, that is characterized by the formation of non-caseating multiple granulomas and is similar in appearance to lesions from tuberculosis, although sarcoid lesions do not contain caseation, typical necrosis or TB bacilli. Giant epithelioid cells, otherwise called Langhan cells, may be numerous or infrequent, but contain intracytoplasmatic inclusions that are not present in tubercolosis and are called Schaumann bodies. These inclusions, however, are not specific for sarcoidosis [7,8]. Diagnosis of neurosarcoidosis depends upon demonstration of a systemic sarcoidosis and the exclusion of other causes for the neurological status. If the lesions of the nervous system do not appear to involve other tissues, as in our case, the diagnosis is misinterpretable and requires histological confirmation [9]. This histological evaluation, in our case, did not show any specific aspects of neurosarcoidosis. Several authors consider the specificity of cerebrospinal fluid angiotensinconverting enzyme (ACE) high enough to warrant inclusion in the diagnostic evaluation of patients in whom CNS neurosarcoidosis is being considered. However the diagnostic accuracy of cerebrospinal fluid ACE is not clearly defined and can not replace the biopsy. ACE was first reported to be increased in CSF in patient with CNS sarcoidosis in the mid-1980s. Currently the discriminator value of 8 nmol/mL/min was associated with the best combination of sensitivity (55%) and specificity (94%) [6,7,9,10] In our case the cerebrospinal fluid ACE activity was 3.2 nmol/mL/min. Futhermore the Kveim test, a specific skin test used to establish the diagnosis of sarcoidosis, wich is usually positive from 60% to 90% depending on the stadium of the desease, in this case was impossible to execute because in our country is not legal.

In addition, non-tumoral intamedullary lesions generally originate from bacterial, fungal or parasytic localizations, but is unusual for an intramedullary abscess to be present so soon in the absence of systemic disease, as in our case. In these cases the most frequent etiopathology of infection depends on intravenous drug use and immune deficiency disorders, aspects that were not present in our patient [11]. The lesion was studied with histochemical techniques, like PAS and Ziehl Neelsen, that excluded the possibility of a bacterial or fungal nature.

### Demyelinating lesions

Another intamedullary lesion that mimicks a tumor can be an MS localization. Isolated spinal cord involvement has been rare and can be the initial manifestation of MS [12]. MS is characterized by numerous areas of demyelination and sclerosis in CNS. Generally, in cases without periferical demyelinating lesions, spinal cord biopsy may be a necessary course of action. The histological specific aspects of MS are demyelinating lesions with aggregates of foamy histiocytes [13]. In our case the possibility of MS lesion was excluded because the lesion did not present these histological aspects and there was no evidence of demyelinating lesions in other districts [14]. Another aspect that has to be analysed is the possibility that this lesion may be an intramedullary localization of a demyelinating disease such as SNM (subacute necrotizing myelopathy) [11]. The intramedullary pathological changes that accompany this disease have been well characterized and consist of demyelination, myelomalacia and necrosis, associated with a proliferation of hyalinized capillary-sized vessels and occasional intraluminal thrombosis and endoluminal calcifications [11], aspects which were not present in our case.

### Degenerative and iatrogenic lesions

Another lesion which may mimick an intramedullary tumor is radiation melyopathy [11]. The most common type of this disease is called Chronic Progressive Radiation Myelopathy CPRM, that usually appears 15-20 months after radiation therapy. The histological pattern is very similar to SNM, with plasmacytic infiltration, necrosis and venous teleangectasias. In our case the patient didn't show these aspects and moreover had never undergone radiation therapy. Furthermore degenerative diseases can mimick intramedullary tumors caused by contrast uptake of the myelopathy; In our case imaging excluded such an origin of the disease.

In our experience it is considered appropriate strategy decompression surgery to be performed as soon as the symptoms given by compression of cord manifested by worsening paraparesis or paraplegia. All of that is supported later by histological analysis that can guide intraoperative tank towards the complete removal or to a simple biopsy then integrated with medical therapy. In our case, the therapeutic strategy "wait and see" was based exclusively on the aggravation of the clinical, especially neurological symptoms, radiological outcome and appearance of the extemporaneous histological lesions that favored an injury repetitive small cell lung cancer. All these aspects justified up to us the complete removal of the lesion, then the result is justified by the complete regression of symptoms.

## Conclusions

Intramedullary lesions that mimick a tumor can be various and difficult to interpret. Preoperative MRI does not allow a certain diagnosis because these lesions have a very similar signal intensity pattern. Specific tests for infective pathologies such as toxoplasmosis and TBC, besides specific tests for sarcoidosis, are useful for diagnosis. Histological examination is often essential for establishing a certain diagnosis. In our case the worsening of symptoms oriented us to a decompressive surgical strategy and total removal of the lesion, also in relation to the extemporary histological examination: this proved correct because of the drastic improvement observed in symptomatology and the total regression, without recrudescence, of symptoms and disease at 12 months follow-up. The final histological examination and the specific tests that we performed have not cleared our doubts regarding the nature of the lesion that remains controversial.

## Consent statement

Written informed consent was obtained from the patient for publication of this case report and any accompanying images. A copy of the written consent is available for review by the Editor-in-Chief of this journal.

## Competing interests

The authors have not been influenced by any financial or personal relationship with people or organizations in preparation of this study.

## Authors' contributions

All authors have made substantial contributions to in the design of the article:

AL was responsible for editing, English editing, correction, search of the literature, conception and design, and has contributed in surgical technique. VDN was responsible for editorship of the manuscript. DED was responsible for the search of the literature. MC was responsible for the search of the literature. RT was responsible for the English editing. MA was responsible for the histology consulting and pathology examination. AS is the principal surgeon and was responsible for editing RD is the principal surgeon and was responsible for editing.
